# Pulmonary blastoma that was first diagnosed as dedifferentiated chondrosarcoma using medical thoracoscopy: A case report

**DOI:** 10.1097/MD.0000000000031377

**Published:** 2022-11-25

**Authors:** Tae Hun Kim, Jae Hyun Jeon, Jin-Haeng Chung, Young-Jae Cho

**Affiliations:** a Divisions of Pulmonary and Critical Care Medicine, Department of Internal Medicine, Seoul National University College of Medicine, Seoul National University Bundang Hospital, Seongnam, South Korea; b Department of Thoracic and Cardiovascular Surgery, Seoul National University College of Medicine, Seoul National University Bundang Hospital, Seongnam, South Korea; c Department of Pathology, Seoul National University College of Medicine, Seoul National University Bundang Hospital, Seongnam, South Korea.

**Keywords:** medical thoracoscopy, pleural effusion, pulmonary blastoma, video-assisted thoracoscopic surgery

## Abstract

Pulmonary blastoma is an extremely rare and highly aggressive tumor. Only a few hundred cases of pulmonary blastoma have been reported. In other cases, a definitive diagnosis is often made through surgical resection. The use of preoperative histopathological sampling in diagnosing was of limited value because of the variety of pulmonary blastoma histology. And there was no literature that the first biopsy was attempted with medical thoracoscopy for diagnosis.

**Patient concerns:**

A 65-year-old man presented to our hospital with pleural effusion and lung mass.

**Diagnoses:**

The patient was initially diagnosed with dedifferentiated chondrosarcoma by medical thoracoscopic biopsy but the final diagnosis was pulmonary blastoma through bilobectomy.

**Interventions:**

Medical thoracoscopy, and video-assisted thoracoscopic surgery (bilobectomy) followed by adjuvant chemotherapy.

**Outcomes:**

After surgical resection of the tumor, adjuvant chemotherapy has been performed 5 cycles at 3 weeks intervals, and there was no evidence of recurrence on follow-up computed tomography performed 4 months after surgery.

**Lessons:**

Medical thoracoscopy is useful for the diagnosis of indeterminate pleural effusion; however, caution is needed when confirming rare malignancies, such as pulmonary blastoma. Although surgical resection is the treatment of choice, appropriate adjuvant chemotherapy to improve the prognosis may be necessary if there is pleural metastasis.

## 1. Introduction

Pulmonary blastoma (PB) is an extremely rare and highly aggressive tumor, constituting approximately 0.5% of all lung tumors.^[1]^ Only a few hundred cases were reported in the literature.^[2,3]^ The pathogenesis of these tumors remains uncertain, but owing to their resemblance to fetal lung tissue, it has been proposed that they originate from pluripotent PB.^[4,5]^ PB usually presents as a mass on chest radiography, as in the present case. The use of preoperative histopathological sampling in diagnosing PB by methods such as needle aspiration was of limited value because the histology of PB varies and is misleading with features resembling adenocarcinoma or another sarcoma of the lung.^[6]^ So in other cases, a definitive diagnosis of PB is often made through surgical resection.^[7]^ But there was no literature that the first biopsy was attempted with medical thoracoscopy (MT) for diagnosis of a PB case. Herein, we report the case of PB treated by bilobectomy which was first diagnosed as dedifferentiated chondrosarcoma using MT.

## 2. Case presentation

Chest radiography in a 65-year-old man with mild dyspnea and right-sided chest discomfort revealed a mass in the right lower lung field. His medical history was uneventful except for previous dyslipidemia. He had a 15-pack-year smoking history but had denied smoking since 2000. He denied alcohol consumption, tuberculosis exposure, occupational exposure to asbestosis or teratogenic materials, or a family history of malignancy.

**Figure 1.**
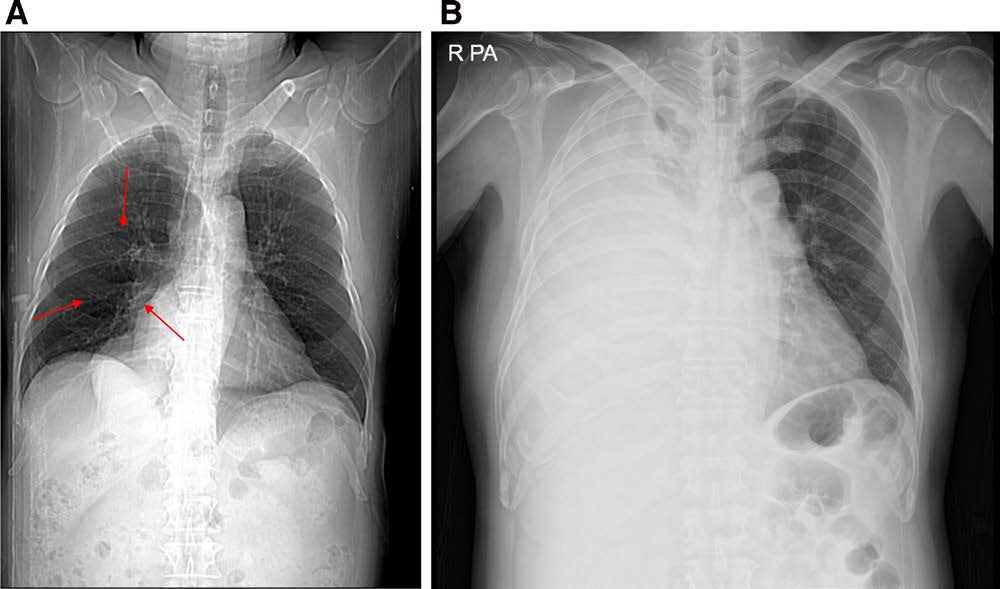
(A) Chest radiograph revealed mass opacities in the right lower lung fields (arrow). (B) Follow-up chest radiography showed a large right pleural effusion with compressive atelectasis.

**Figure 2.**
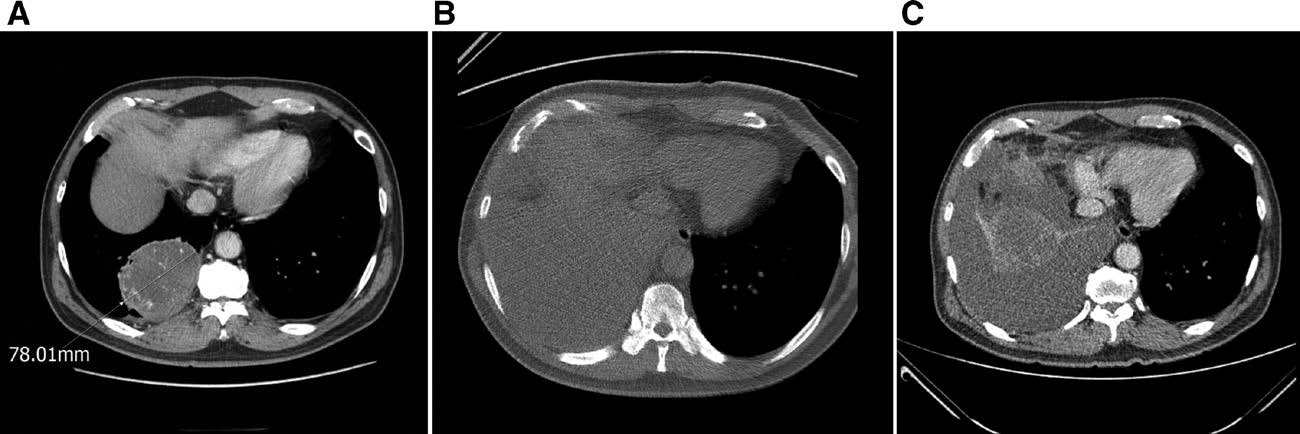
(A) CT scan of the thorax revealed a right lower lung mass. (B). There were large amounts of pleural effusion and target mass hard to distinguish on CT performed during a lung biopsy attempt. (C) There was a large right pleural effusion with compressive atelectasis and mass-like opacity at follow-up enhanced chest CT. CT = computed tomography.

Chest radiography revealed mass opacities in the right lower lung fields (Fig. 1A). Chest computed tomography (CT) performed at a local medical center revealed a right lower lung mass (78 mm) (Fig. 2A). We initially planned to perform a CT-guided percutaneous lung biopsy. However, the patient was quarantined with COVID-19. When CT-guided percutaneous lung biopsy was attempted after release from isolation, there was a large pleural effusion, and the mass for biopsy could not be distinguished on the scanned CT image (Fig. 2B). Biopsies could not be performed.

We performed diagnostic and therapeutic thoracentesis and planned an enhanced chest CT tracing. Pleural effusion analysis showed lymphocyte-dominant exudate, and the results were as follows: protein > 5000 mg/dL, LDH 553 U/L, pH 7.555, glucose 231 mg/dL, polymorphonuclear cell: 12%, mononuclear cell: 88%, ADA 19 IU/L, negative for AFB, negative for MTB PCR, and no microorganism was isolated from the culture specimen. Cytological tests also reported non-diagnostic, benign cellular changes without malignant cells. Follow-up chest radiography (Fig. 1B) and chest CT (Fig. 2C) were performed 1 week later and showed a large right pleural effusion with compressive atelectasis.

**Figure 3.**
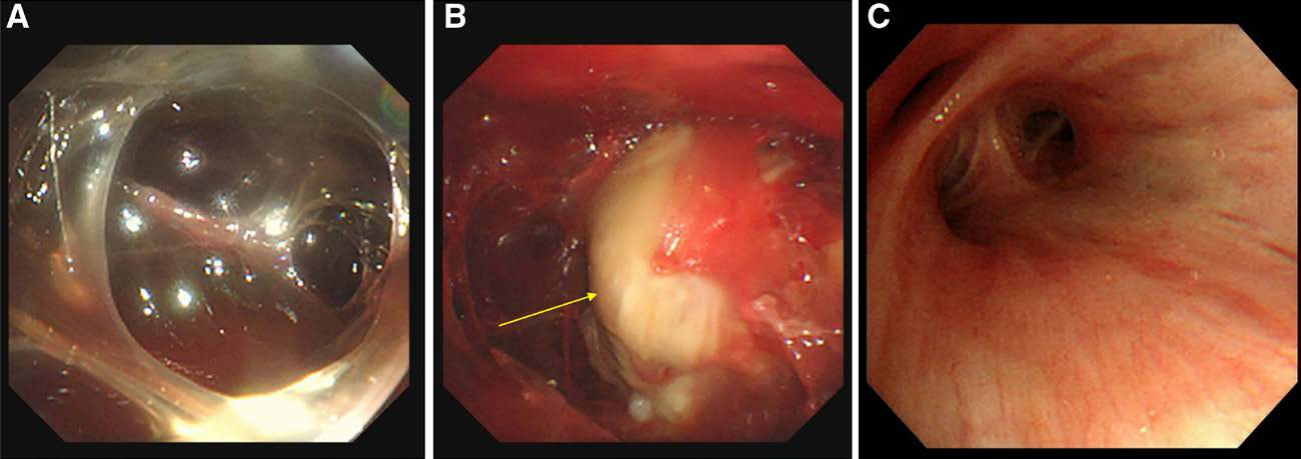
(A) Medical thoracoscopic view showed separated pleural space and severe adhesion of the pleura. (B) Whitish mass (arrow) which was connected to the lung basal to pleura. (C) Bronchoscopic view at the level of the right bronchus intermedius.

The patient was admitted to the emergency room and underwent percutaneous catheter drainage of the right lung. MT was performed as the diagnostic method. In MT, the pleural space was separated, and severe adhesion of the pleura was observed (Fig. 3A). As many pleural biopsies as possible were performed. A whitish mass was observed, which was connected to the lung base and pleura (Fig. 3B). The white mass was hard and was therefore difficult to biopsy using forceps. Bronchoscopy was also performed, but there was an external collapsed bronchus without an endobronchial tumorous lesion in the right lower bronchus (Fig. 3C).

**Figure 4.**
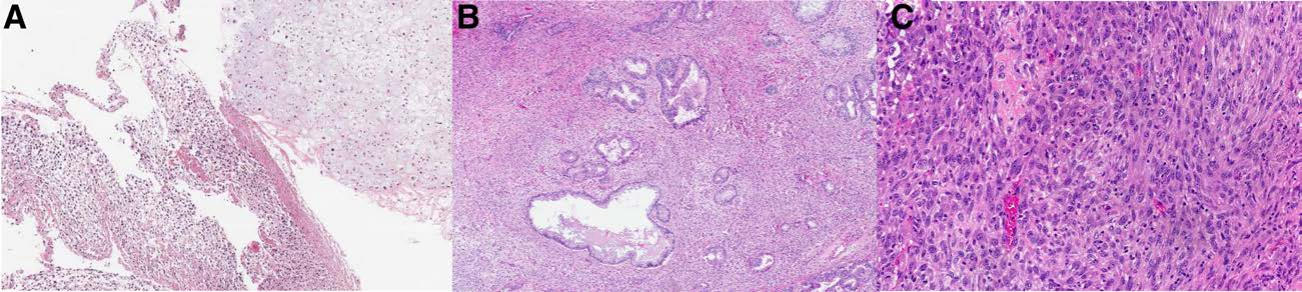
(A) Medical thoracoscopic biopsy showed a malignant mesenchymal tumor composed of atypical cartilage and undifferentiated components with necrosis (hematoxylin-eosin, original magnification ×200). (B) Histopathology of fetal-type glandular structures mixed with mesenchymal component showing a tendency to differentiate toward more mature fibroblast-like cells (hematoxylin-eosin, original magnification ×100). (C) The primitive blastemal component with heterologous chondrosarcoma element (inset) (hematoxylin-eosin, original magnification ×200).

The histology of the white mass revealed malignant mesenchymal tumor cells (Fig. 4A). Immunohistochemically, the tumor was positive for S-100 and cluster of differentiation CD99 in the sarcomatous component, but negative for CD 34, calretinin, STAT6, D2-40 (podoplanin), and cytokeratin. Pleural tissue showed chronic inflammation and was negative for malignancy. Brain magnetic resonance imaging and positron emission tomography - computed tomography confirmed no other metastatic lesions, except lung lesions. We decided to perform surgical tumor resection by a multidisciplinary team.

**Figure 5.**
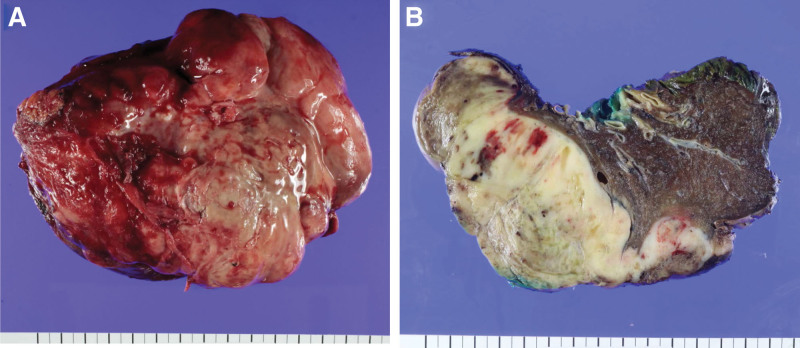
(A and B) Resected mass specimen of right lower and right middle lung through exploratory thoracotomy.

An exploratory thoracotomy was performed. A pleural frozen biopsy was performed at 5 sites through surgery, and a large mass originating from the right lower lung adjacent to a minor fissure was observed, which could not be fully dissected; thus, bilobectomy was performed (Fig. 5A and B). The size of the resected right lower and middle lung mass was 13.7 cm × 12.3 cm × 5.5 cm and 13.7 cm × 8.7 cm × 5.4 cm, respectively. The pathologic diagnosis was PB composed of areas of epithelial and mesenchymal differentiation. (40% primitive mesenchymal area, 50% chondrosarcoma, 5% and 5% osteosarcoma). The epithelial component was low-grade fetal-type adenocarcinoma, consisting of branching tubules with clear to weakly eosinophilic cytoplasm. The columnar cells resemble the fetal lung in the pseudoglandular period (Fig. 4B). The mesenchymal component showed tightly packed primitive oval cells with a high nuclear-to-cytoplamsic ratio, and heterologous components, such as chondrosarcoma and osteosarcoma (Fig. 4C). Immunohistochemically, the primitive mesenchymal cells were positive for CD99 and cytokeratin and TTF-1 were expressed only in carcinomatous area. Both glandular and blastematous components showed nuclear positivity of ß-catenin. As pleural metastasis was confirmed by biopsy, final pathologic stage was pT4N0M1, R2 resection.

Three weeks after discharge, he visited an oncologist and started adjuvant chemotherapy with etoposide, ifosfamide, and cisplatin. To date, chemotherapy was performed 5 cycles at 3 weeks intervals, and there was no evidence of recurrence on follow-up CT performed 4 months after surgery.

## 3. Discussion

PB is an extremely rare and highly aggressive tumor, constituting approximately 0.5% of all lung tumors.^[1]^ Preoperative biopsy could be attempted but caution is needed when confirming rare malignancies with various histological types including PB. Surgical resection is the treatment of choice for PB. However, the prognosis of PB is considered poor. The 5-year survival rate for PB is documented at 16% to 25%, and 10-year at 8%.^[8]^ And the 5-year overall survival for patients with localized stage was more than 75%, while for patients with regional or distant stage was less than 40%.^[3]^ Therefore, surgical resection that does not leave residual mass is important for treatment. But local recurrence occurs in 43% after surgical resection, and regional and distant metastases are frequently reported.^[9,10]^

Chemotherapy and radiotherapy also have been used for the patients with advanced stage. But no standard treatment guidelines have been established for inoperable cases. Chemotherapy regimens selected include various combinations of agents like cisplatin, etoposide, ifosfamide, mitomycin C, and pemetrexed.^[11]^ In this case, adjuvant chemotherapy with etoposide, ifosfamide, and cisplatin has been performed because post-operative final pathologic stage was pT4N0M1 with R2 resection.

## 4. Conclusion

We present the diagnosis process of the case of PB, a rare pulmonary malignancy. And the patient treated with surgical resection and adjuvant chemotherapy. This case is interesting in that the first biopsy attempt was done through a MT and the lesson was learned to be caution is needed when confirming rare malignancies, such as PB. Although efforts have been made for complete resection with wide surgical margin excision, appropriate chemotherapy to improve the prognosis may be necessary if there is pleural metastasis as in this case.

## Author contributions

**Conceptualization:** Tae Hun Kim, Jae Hyun Jeon, Jin-Haeng Chung, Young-Jae Cho.

**Data curation:** Tae Hun Kim, Jin-Haeng Chung.

**Investigation:** Tae Hun Kim, Jae Hyun Jeon, Young-Jae Cho.

**Supervision:** Jae Hyun Jeon, Jin-Haeng Chung, Young-Jae Cho.

**Writing – original draft:** Tae Hun Kim.

**Writing – review & editing:** Tae Hun Kim, Jae Hyun Jeon, Jin-Haeng Chung, Young-Jae Cho.
